# The protective effect of fermented *Curcuma longa* L. on memory dysfunction in oxidative stress-induced C6 gliomal cells, proinflammatory-activated BV2 microglial cells, and scopolamine-induced amnesia model in mice

**DOI:** 10.1186/s12906-017-1880-3

**Published:** 2017-07-17

**Authors:** Cheong-Su Eun, Jong-Soon Lim, Jihye Lee, Sam-Pin Lee, Seun-Ah Yang

**Affiliations:** 1 Major in Food Science and Technology, Keimyung University, Daegu, 42601 Republic of Korea; 2 The Center for Traditional Microorganism Resources, Keimyung University, Daegu, 42601 Republic of Korea; 3 Department of Biomedical Science, Graduate School, Kyungpook National University, Daegu, 38578 Republic of Korea

**Keywords:** Fermented *Curcuma longa* L, Memory dysfunction, C6 glioma cells, BV2 microglial cells, Scopolamine-induced amnesia model

## Abstract

**Background:**

*Curcuma longa* L. is a well-known medicinal plant that has been used for its anti-cancer, neuroprotective, and hepatoprotective effects. However, the neuroprotective effect of fermented *C. longa* (FCL) has not been reported. Therefore, in this study, the effectiveness of FCL for the regulation of memory dysfunction was investigated in two brain cell lines (rat glioma C6 and murine microglia BV2) and scopolamine-treated mice.

**Methods:**

*C. longa* powder was fermented by 5% *Lactobacillus plantarum* K154 containing 2% (*w*/*v*) yeast extract at 30 °C for 72 h followed by sterilization at 121 °C for 15 min. The protective effects of fermented *C. longa* (FCL) on oxidative stress induced cell death were analyzed by MTT assay in C6 cells. The anti-inflammatory effects of FCL were investigated by measuring the production of nitric oxide (NO) and prostaglandin E_2_ (PGE_2_) as well as the expression levels of inducible NO synthase (iNOS) and cyclooxygenase-2 (COX-2) in LPS-stimulated BV2 cells. The step-through passive avoidance test, Morris water maze test, acetylcholinesterase (AChE) activity, and expression of cAMP response element-binding protein (CREB) and brain-derived neurotropic factor (BDNF) were employed to determine the effects of FCL on scopolamine-induced memory deficit in mice. The contents of curcuminoids were analyzed through LC/MS.

**Results:**

Pretreatment with FCL effectively prevented the cell death induced by oxidative stress in C6 cells. Moreover, FCL inhibited the production NO and PGE_2_ via the inhibition of iNOS and COX-2 expression in BV2 cells. FCL significantly attenuated scopolamine-induced memory impairment in mice and prevented scopolamine-induced AChE activity in the hippocampus. Additionally, FCL reversed the reduction of CREB and BDNF expression. The curcuminoids content in FCL was 1.44%.

**Conclusion:**

FCL pretreatment could alleviate scopolamine-induced memory impairment in mice, as well as oxidative stress and inflammation in C6 and BV2 cells, respectively. Thus, FCL might be a useful material for preventing impairment of learning and memory.

## Background

Cognitive impairment, declining learning ability and memory, are common symptoms in age-related neurodegenerative diseases including Alzheimer’s disease (AD), Parkinson’s disease, and stroke. AD is the most common cause of dementia, accompanied by the deposition of amyloid plaques and neurofibrillary tangles [1, 2]. In normal brain function, glial cells, including astrocytes and microglial cells, play critical roles in supporting and protecting neurons. Glial cells are abundant in the hippocampus and cortex, which control the ability for learning and memory. When glial cells (e.g., rat glioma C6, microglial BV2) are activated by free radicals, inflammatory process is initiated, resulting in the death of neuronal cells by the production of pro-inflammatory and neurotoxic factor such as inducible nitric oxide synthase (iNOS), cyclooxygenase-2 (COX-2), and several cytokines [3, 4]. C6 and BV2 cells have been extensively used as cell models of neurotoxicity to investigate responses to pro-inflammatory cytokines, lipopolysaccharide (LPS), and oxidative stress for searching new drug candidates [5, 6].

Recent studies have suggested that the brain levels of acetylcholine (ACh), a key neurotransmitter for normal cognitive function, are increased through inhibition of its metabolizing enzyme, acetylcholinesterase (AChE) [7, 8]. Thus, AChE inhibitors such as donepezil increase cholinergic activity to relief the cognitive symptoms of AD.

Administration of scopolamine, a potent amnestic agent, in animals blocked the central muscarinic acetylcholine receptor and impaired learning and memory functions [9]. The scopolamine-induced amnesia model is commonly used for screening memory enhancing drugs. Moreover, previous genetic studies have demonstrated that the activation of the cAMP-response element binding protein (CREB) plays a critical role by turning on the molecular switch of the brain-derived neurotrophic factor (BDNF) for long-term memory formation [10, 11]. At the same time, BDNF production is also induced by the activation of CREB. A decline in BDNF levels is observed in patients with AD [12], suggesting that CREB signaling and BDNF expression are associated with normal cognitive function.

*Curcuma longa* L. (Zingiberaceae family) has been widely used as a folk medicines as well as a spice in Asia, especially in India, and curcuminoids such as curcumin, demethoxycurcumin (DMC), and bisdemethoxycurcumin (BDMC) are known to be the major components present in its rhizome [13]. Curcumin is the most well-known and extensively studied curcuminoids for its anti-oxidant, anticancer, anti-inflammatory, neuroprotective, and hepatoprotective properties [14–16]. Although *C. longa* has various pharmaceutical properties, little information is available regarding the effects of fermented *C. longa* (FCL) in improving memory and treating neurodegenerative conditions.

The present study was therefore conducted to reveal the in vitro protective effects of FCL against tert-buryl hydroperoxide (t-BHP)- and hydrogen peroxide (H_2_O_2_) in C6 cells and LPS in BV2 cells, as cell models of memory impairment. In addition, the in vivo protective effects of FCL were investigated in a scopolamine-induced amnesia animal model by behavioral and immunohistochemical analysis. We also evaluated the amount of curcuminoids (curcumin, DMC, and BDMC) in FCL by liquid chromatography-tandem mass spectrometry (LC-MS/MS). To the best of our knowledge, this is the first report of the effectiveness of FCL against neurodegenerative conditions in vitro and in vivo.

## Methods

### Chemicals and reagents

Fetal bovine serum (FBS), dulbecco’s modified eagle medium (DMEM), and other cell culture reagents were obtained from Gibco BRL (Grand Island, NY). Earle’s basal salt solution (EBSS), trypsin solution, 3-(4,5-dimethylthiazol-2-yl)-2,5-diphenyl tetrazolium bromide (MTT), t-BHP, H_2_O_2,_ LPS, (−) scopolamine hydrobromide, acetylthiocholine iodide, 5,5-dithiobis[2-nitrobenzoic acid] (DTNB) were purchased from Sigma Chemical Co. (St. Louis, MO). Primary antibodies specific for iNOS, COX-2, β-actin, BDNF, pCREB and rabbit secondary antibody were purchased Cell Signaling Technology (Danvers, MA). Glyceraldehyde-3-phosphate dehydrogenase (GAPDH) and CREB antibodies were purchased from Santa Cruz Biotech (Santa Cruz, CA).

### Preparation of FCL

Dry powder of *C. longa* L. (Myanma) was purchased from the medicinal herb market (www.hanyakjae.net) (Seoul. Korea). A voucher specimen was deposited at the Natural Product and Food Research Laboratory, Keimyung University, Daegu, Korea. To prepare FCL, *C. longa* powder was fermented by 5% *Lactobacillus plantarum* K154 [17] containing 2% (*w*/*v*) yeast extract without monosodium glutamic acid at 30 °C for 72 h followed by sterilization at 121 °C for 15 min. *C. longa* powder and FCL were extracted with 70% ethanol and the lyophilized extracts were stored at −20 °C until used.

### Cell culture

C6 rat glioma cells were purchased from the Korean Cell Line Bank (KCLB, Seoul, Korea) and cultured in DMEM (Gibco BRL, Grand Island, NY) with 10% FBS (Gibco BRL, Grand Island, NY) and 1% streptomycin/penicillin in a 37 °C humidified incubator in an atmosphere of 5% CO_2_ in air. BV2 murine microglial cells were provided by Prof. G.S. Jeong (Dept. Pharmaceutics, Keimyung University, Daegu, Korea) and cultured in α-MEM supplemented with 10% FBS containing 100 U/ml of penicillin and 100 μg/ml of streptomycin at 37 °C in a 5% CO_2_ humidified incubator.

### Cell viability

Cell viabilities were determined by the MTT assay [18]. C6 cells (5 × 10^4^ cells /well) were seeded in a 96-well plate and pretreated with various concentrations of FCL for one day. The cells were incubated with t-BHP (1 mM) for 1 h and H_2_O_2_ (2 mM) for 30 min to induce oxidative stress. BV-2 cell were incubated in 96-well plates at a density of 1 × 10^5^ cells per wells and pretreated with various concentration of FCL for 1 h and then stimulated by LPS (100 ng/ml) for an additional 24 h in the presence of FCL. Following treatment, 10 μl of a MTT solution (5 mg/ml in phosphate buffered saline) was added to each well and further incubated for 4 h at 37 °C. Subsequently, 100 μl of dimethyl sulfoxide (DMSO) was added to each well to solubilize any deposited formazon. The optical density of each well was measured at 550 nm with a microplate reader (Molecular Devices, Spectra max 340PC, USA).

### Assay for nitric oxide (NO), prostaglandin E2 (PGE2), tumor necrosis factor α (TNF-α)

NO production in culture medium was assayed via the Griess Reagent System [19]. The culture supernatant (100 μl) was mixed with same volume of Griess reagent (1% sulfanilamide, 0.1% naphthylethylendiamine in 2.5% phosphoric acid) in a 96-well plate. After an incubation of 10 min at room temperature, the optical density was determined at 540 nm with a microplate reader. Levels of PGE_2_ in the media were measured using an immunoenzymatic method (Cayman Chemicals, San Diego, CA) according to the manufacturer’s specifications. BV2 microglia (1 × 10^5^ cells per well) were pretreated with FCL for 1 h and stimulated with LPS (100 ng/ml) for an additional 24 h in the presence of FCL. The PGE_2_ level in the supernatants (50 μl) was estimated using a specific enzyme immunoassay kit. TNF-α in the supernatants and in the medium were assessed with commercially available ELISA kits (PEPROTECH. Rocky Hill, NJ), according to the manufacturer’s instructions. The ELISA assay was performed in triplicate.

### Animals

Male ICR mice (OrientBio, GyeongGi-Do, Korea), weighing 23–25 g at the beginning of the experiments, were used. The animals were housed 8 per cage and maintained in temperature (23 ± 2 °C) and humidity (60 ± 10%) under a 12-h light/12-h dark cycle (08:00–20:00 h lights on) with food and water available ad libitum. The mice were maintained under laboratory conditions for an acclimatization period of 7 days before performing the tests. This procedure was approved by the Animal Care and Use Committee of Daegu Haany University (DHU 2013–070).

### FCL administration

FCT was suspended in 10% Tween 80 solution for use. Donepezil and scopolamine were dissolved in saline. The mice were randomly divided into six groups of eight individuals as follows: control group (*n* = 8), the vehicle solution-treated and scopolamine-induced group (*n* = 8), the FCL-treated and scopolamine-induced groups (50, 100, or 200 mg/kg, *p.o., n* = 8), and donepezil-treated (5 mg/kg, *p.o.*, *n* = 8) and scopolamine group (1 mg/kg, i.p., *n* = 8). In the scopolamine-induced memory impairment, FCT or donepezil were given 1 h before the acquisition trial in the passive avoidance test, and 1 h before the first trial session every consecutive day in the water maze task. In the control group, vehicle solution (10% Tween 80, *p.o.*) was administered using the same time schedule. Memory impairment was induced by scopolamine treatment 30 min before each test.

### Step-through passive avoidance test

Acquisition and retention trials of step-through passive avoidance test were conducted in identical light and dark boxes (Gemini Avoidance System, San Diego, CA). The light compartment (20 × 20 × 20 cm) contained a 50 W bulb, and its floor was composed of 2 mm steel rods spaced 1 cm apart. The floor of dark compartment also consisted of 2 mm steel rods spaces 1 cm apart, as previously described [20]. These compartments were separated by guillotine door (5 × 5 cm). Mice underwent two separated trials, an acquisition trial and a retention trial 24 h later. One hour before the acquisition trial, the mice were orally administrated FCL (50, 100, 200 mg/kg, *p.o.*), donepezil (5 mg/kg) as a positive drug, or same amount of vehicle solution. Memory impairment was induced by scopolamine (1 mg/kg, i.p.) 30 min prior to the acquisition trial. For the acquisition trial, each mouse was placed in the light compartment, and the guillotine door was opened 10 s later. When a mouse entered the non-illuminated dark compartment, the guillotine door automatically closed and an electrical foot shock (0.5 mA, 3 s) was delivered through the floor rods. The retention trial was carried out 24 h after the acquisition trial. The mouse were again placed in the light compartment, and the time for each mouse to enter the non-illuminated compartment after door opening was recorded as latency times in both trials. Latency time was measured for up to 300 s.

### Morris water maze test

Morris water maze was consisted of a circular pool (diameter 90 cm, height 45 cm) filled with water containing black pigment, and the testing procedure was same as that described previously by Morris [21]. A platform (diameter 60 cm, 1 cm below the water surface) was then placed in one of the pool quadrants. Animals were gently plunged into the water pool with its face toward the wall of the pool in one of the pool quadrants. The entry point was changed in a different order each day. During the 4 subsequent days, mice were allowed to swim the pool in search of the escape platform and recorded the time using video camera-based Ethovision software (Noldus, Netherlands). In the last trial, mice were subjected to probe trial without platform for 120 s. A record was kept of the swimming time in the pool quadrant where the platform originally had been located.

### AChE inhibition assay

AChE activity was evaluated using mice brain supernatants on the basis of the colorimetric method [22]. Whole brains of male ICR mice were homogenized in a glass Teflon homogenizer (Eyela, Japan) containing 10 volumes of homogenizer buffer (0.1 M sodium phosphate buffer, pH 8.0) and then centrifuged at 14,500 x rpm for 20 min at 4 °C. The supernatants were collected and used as the enzyme source for the assay. FCL and donepezil were dissolved in DMSO and diluted to various concentrations immediately before use. A mixture of diluted sample or drug solution (10 μl), substrate (5 μl acetylthiocholine iodide), Ellman’s reagent (25 μl 5,5′-dithiobis-2-nitrobenzoic acid in 0.1 M phosphate buffer, pH 7.0) and 0.1 M phosphate buffer (640 μl) was incubated for 30 min at room temperature. The enzyme source was added to this mixture, which was further incubated for 1 min. Absorbance was measured at 410 nm and the concentration of sample required to inhibit acetylcholinesterase activity by 50% (IC_50_) was calculated using an enzyme inhibition dose response curve.

### Western blotting

Western blot analysis was performed as described previously [23] with some modification. BV-2 cells were plated at a density of 1 × 10^6^ cells per ml in a 6-well cell culture plate with 2 ml of culture medium and incubated for 24 h. The cells were pre-treated with FCL for 1 h and stimulated with LPS (100 ng/ml) for specified time periods. Then, cells were harvested on ice and by scraping the cells from cultured dishes using a cell EDTA and were collected. The cells were washed with phosphate-buffered saline (PBS) and lysed with lysis buffer (2 mM EDTA, 100 mM NaCl, 0.5% Triton X-100, 2 mM PMSF, 10 mM sodium orthovanadate, 2 ng/L leupeptin, 1 μg/mL aprotinin, in 50 mM Tris-HCl, pH 7.5). Lysates were then centrifuged at 12,000 x rpm at 4 °C. To investigate the effects of FCL on pCREB and BDNF expression in the hippocampus, isolated hippocampal tissues were homogenized with RIPA Buffer (Cell Signaling, Danvers, MA) and centrifuged for 20 min at 14,500 x rpm at 4 °C. Proteins (20 μg) were separated in sodium dodecyl sulfate (SDS)-polyacrylamide gels and transferred to polyvinylidene fluoride (PVDF) membrane. Membranes were blocked in 5% skim milk in Tris-buffered saline with 0.1% Tween-20 (TBST) for 1 h and then incubated overnight at 4 °C with primary antibodies (1:1000 dilution). Membranes were rinsed three times in TBST and incubated 1 h at room temperature with secondary antibodies (1:2000 dilution). The membranes were rinsed and developed by chemiluminescence and visualized using an ImageQuant LAS 4000 mini (GE Healthcare, NJ). The values were normalized by taking ratio of BDNF and pCREB against GAPDH and CREB respectively, to correct for any loading and transfer differences between samples.

### Immunohistochemistry

For immunohistochemical studies, mice were anesthetized with zoletil and then perfused with 4% formaldehyde dissolved in 0.1 M PBS (pH 7.4), as described previously [24] with some modification. Brains were removed and post-fixed in 4% paraformaldehyde (PFA) diluted in 0.1 M PBS for 24 h and immersed in 30% sucrose solution. Serial paraffin sections (30 μm) were obtained from paraffin embedded brain blocks, and deparaffinized and rehydrated through a series of graded alcohols. After washing with 0.1 M PBS, sections were treated with 0.01 M citric acid (pH 6.0) for 15 min in a microwave for antigen retrieval. To quench endogenous peroxidase activity, the sections were washed again and incubated in 0.3% H_2_O_2_ in 0.1 M PBS for 30 min at room temperature. Sections were immersed for 1 h in blocking solution (0.1% Triton X-100, 1% BSA, and 5% serum in PBS), and incubated with anti-pCREB (1:500 dilution) or BDNF (1:50 dilution) antibody in blocking solution at 4 °C overnight. The sections were then incubated with biotinylated secondary antibodies (1:200 dilution) for 1 h at room temperature. To visualize immunoreactivity, the sections were treated with avidin-biotin complex (ABC) reagents (ABC kit universal; Vector Labs. Co.) for 1 h at room temperature, and incubated with 3,3-diaminobenzidine tetrahydrochloride (DAB) and 0.01% H_2_O_2_ for 3 min. After rinsing with distilled water, sections were dehydrated using an ethanol series followed by xylene and mounted. Histological images were observed under the microscope (Leica Microsystems DM2500/DFC450C, Wetzlar, Germany).

### LC-MS/MS analysis

LC-MS/MS was performed with an Agilent 6410 Triple Quad, tandem mass spectrometry (Agilent Technologies, Palo Alto, CA). The mass spectrometer was operated in ESI positive selected ion monitoring (SIM) mode. The ionization conditions were adjusted at 350 °C and 4 kV for capillary temperature and voltage, respectively. The nebulizer pressure was 40 psig, and the nitrogen flow rate was 12 L/min. The column was a Holo C18 (2.1 × 150 mm, 2.7 um). The mobile phase consisted of A: 0.1% formic acid in H_2_O B; 0.1% formic acid in ACN with a flow rate of 0.4 mL/min. The gradient elution conditions were as follows: 0–5 min, 5–15% B; 5–10 min, 15–30% B; 10–15 min, 30–60% B; 15–20 min, 60–100% B; 20–25 min, 100% B.

### Statistical analysis

All experiments were performed at least in triplicate. Data were expressed as the mean ± standard error of the mean (SEM) or standard deviation (SD). Significant differences from the respective controls for each experimental test condition were assessed using the Student’s *t* test for each paired experiment. Two-way repeated measures analysis of variance (ANOVA) was used to analyze the escape latencies in the Morris water maze test. In the passive avoidance test and AChE inhibition assay, data were analyzed by one-way ANOVA followed by the Student-Newman-Keuls test for multiple comparisons. Statistical significance was set at *P* < 0.05.

## Results and discussion

### Neuroprotective effects of FCL in t-BHP and H_2_O_2_-treated C6 cells

**Fig. 1:**
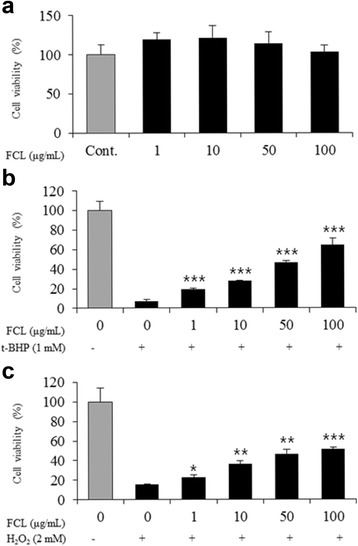
Protective effects of FCL against oxidative stress in C6 cells. Effects on the cell damage induced by t-BHP (**b**) and H_2_O_2_ (**c**) were measured by the MTT assay. Cells were treated with t-BHP (1 mM) for 1 h or H_2_O_2_ (2 mM) for 30 min after the incubated with FCL for 15 h. Data represent means ± SEM of three independent experiments. * *P* < 0.05 ** *P* < 0.01 as compared with the t-BHP- or H_2_O_2_-treated group

To examine the protective potential of FCL against oxidative stress in vitro, its inhibitory effects on t-BHP- and H_2_O_2_-induced cell death were measured in C6 glial cells. As shown in Fig. 1a, FCL showed no cytotoxicity in C6 cells by MTT assay (*P* > 0.05). The induction of cytotoxicity by t-BHP (1 mM) for 1 h in C6 cells induced cell death in about 93% of the cells. The t-BHP-induced cell death was prevented by FCL in a dose-dependent manner (Fig. 1b, *P* < 0.001). Furthermore, cell death increased by about 84% following H_2_O_2_ treatment (2 mM) for 30 min. Pretreatment with FCL effectively prevented the cell death induced by H_2_O_2_ (Fig. 1c, *P* < 0.05).

The role of astrocyte-like C6 glial cells has been widely investigated in maintaining cognitive function under oxidative stress conditions. C6 glial cells are stimulated quickly by external stimuli such as H_2_O_2_ and t-BHP, which induce oxidative stress and cell injury in vitro [25, 26].

### Anti-inflammatory effects of FCL in LPS-stimulated BV2 cells

**Fig. 2:**
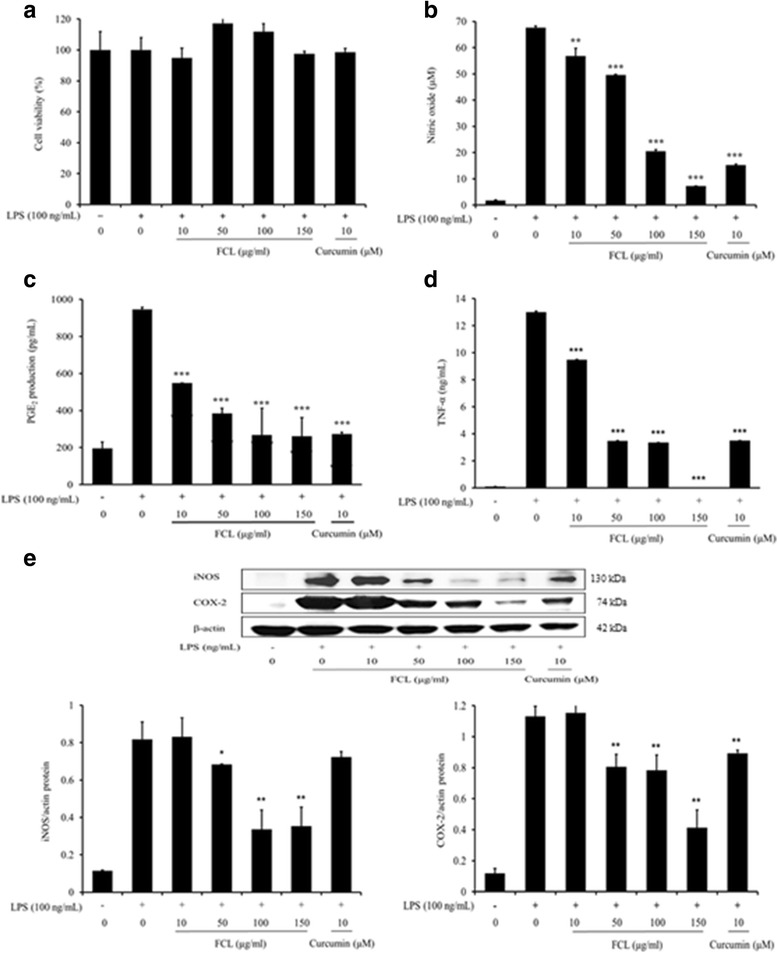
Effects of FCL on cell viability and LPS-induced NO, PGE_2_, TNF-α production and iNOS, COX-2 expressions in BV2 cells. Cells were pretreated with various concentrations of FCL for 1 h and stimulated by LPS (100 ng/mL) for an additional 24 h. Cell viability (**a**) was measured by the MTT assay. NO production (**b**) was assayed in the stimulated cell culture media. Cytokine level was evaluated by enzyme-linked immunosorbent assay for PGE_2_ (**c**), TNF-α (**d**). Total cell lysates (20 μg) were examined for iNOS (130 kDa) and COX-2 (74 kDa) protein expressions by Western blotting, and the relative expression levels were normalized by an internal control, β-actin (42 kDa) (**e**). Data present means ± SD in triplicate. * *P* < 0.05, ** *P* < 0.01, *** *P* < 0.001 vs LPS alone

We investigated the effects of FCL on the production of NO in on LPS-activated BV2 cells. The cells were pretreated with FCL for 1 h and incubated with LPS (100 ng/ml) for an additional 24 h. The cytotoxicity of FCL at concentrations ranging from 10 to 150 μg/ml was tested on BV2 cells by MTT assay. FCL had no effect on BV2 cell viability (*P >* 0.05), as shown in Fig. 2a. Curcumin (10 μM), a reference compound, was also not cytotoxic (*P >* 0.05). To investigate the effects of FCL on neuroinflammation, FCL at various concentrations were was tested for their inhibitory activities against NO production in LPS-stimulated BV2 cells by using Griess reagent. As shown in Fig. 2b, the NO level in the culture supernatant was dramatically increased to 67.6 μM by LPS (100 ng/ml) stimulation. However, FCL (150 μg/ml) suppressed this NO production by up to 91.64% (*P* < 0.001). By comparison, 150 μg/ml of curcumin reduced the NO release by about 76.9% (*P* < 0.001). We next measured the inhibitory activities of FCL on pro-inflammatory mediator PGE_2_ secretion in BV2 cells activated by LPS. FCL significantly decreased the levels of PGE_2_ production in LPS-stimulated cells in a concentration-dependent manner (Fig. 2c, *P* < 0.001)). LPS stimulation caused a substantial secretion of TNF-α; however, pretreatment with FCL dose-dependently suppressed TNF-α production, with 150 μg/ml leading to a reduction to undetectable levels (Fig. 2d, *P* < 0.001)). Treatment with curcumin (10 μM) as a reference significantly reduced both PGE_2_ and TNF-α production (*P* < 0.001). We further investigated the protein expression of iNOS and COX-2, which produce NO and PGE_2_ in BV2 cells. As shown in Fig. 2e, FCL significantly decreased the levels of iNOS and COX-2 proteins in a dose-dependent manner for concentration over 50 μg/ml (*P* < 0.05). Curcumin (10 μM) also significantly inhibited the COX-2 protein expression (*P* < 0.05).

Inflammation has a primary role in the brain aging and chronic neurodegenerative diseases, including Alzheimer’s, Parkinson’s, and Huntington’s disease. Although the activation of microglia is the resident innate immune defense in the central nervous system (CNS) [27], over-activation of microglial cells can cause the inflammatory responses, which produces neurotoxic compounds including NO, PGE2, and TNF-α [28]. Thus, blocking the microglial over-activation could be a reasonable strategy to inhibit toxic pro-inflammatory cytokines-mediated neurodegenerative damage.

Curcumin was shown to suppress LPS-induced COX-2 expression in BV2 cells through the inhibition of activator protein 1 (AP1) and NF-κB binding [29]. It was also suggested that curcumin is a promising dietary agent in the prevention and treatment of microglial cell-mediated neurodegenerative conditions because of the blockade of microglial activation [30, 31]. Zhang et al. [32] also showed that DMC exerts anti-inflammatory effects in LPS-activated N9 microglial cells via blocking activation of NF-κB and mitogen-activated protein kinases (MAPKs).

### Effects of FCL on scopolamine-treated mice in behavioral tests

**Fig. 3:**
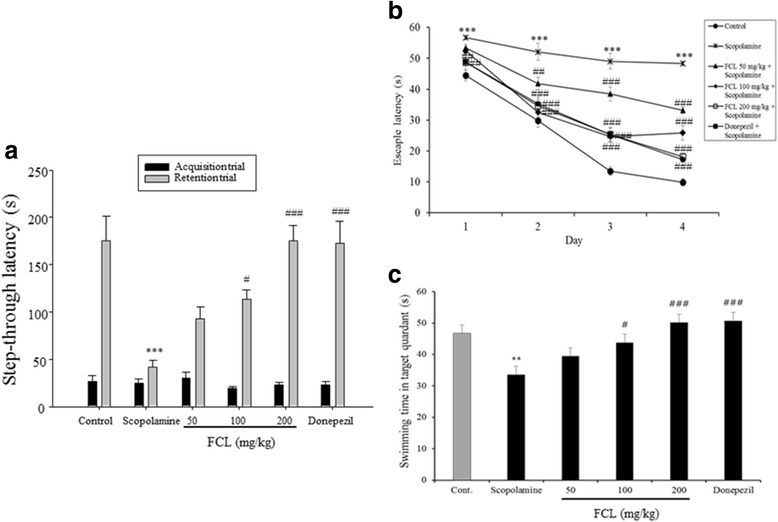
Effects of FCL on scopolamine-induced memory deficit in step-through passive avoidance test (**a**), and escape latency during the training sessions (**b**) and swimming time during the probe trial session of Morris water maze test during four days (**c**).FCL or donepezil (5 mg/kg, p.o.) was administrated 60 min prior to the acquisition trial or the first training trial of each training day. Scopolamine (1 mg/kg, i.p.) was injected 30 min after the drug administration. Data represent means ± SEM (*n* = 8). ** *P* < 0.01 *** *P* < 0.001 as compared with the control group. # *P* < 0.05, ## *P* < 0.01 and ### *P* < 0.001 as compared with the scopolamine-treated group

The effects of FCL on scopolamine-induced memory impairment were investigated using the step-through passive avoidance test and the Morris water maze test. Scopolamine is a non-selective muscarinic acetylcholine receptor antagonist that induces cognitive impairment in animal models [9]. Thus, the scopolamine-induced memory deficits animal model has been widely used for screening anti-amnesia drugs. The step-through latency test demonstrates deficits in cognitive and long-term memory [33, 34]. As shown in Fig. 3a, no significant differences were observed in the step-through latency times of the acquisition trials (training day 0) among the groups [F(5,36) = 0.831, *P* > 0.05]. In the retention trial after 24 h of the acquisition trial, we observed that the step-through latency time to reenter the dark compartment was significantly decreased by a single administration of scopolamine (1 mg/kg, i.p.) when compared to the control group mice (*P* < 0.001). The pretreatment of FCL (100 and 200 mg/kg, *p.o.*) or donepezil (5 mg/kg, *p.o.*) showed a significant group effect on the step-through latency in the retention trial [F(5,36) = 12.80, *P <* 0.001]. In this study, donepezil, an acetylcholinesterase inhibitor, was used as a positive control.

We next conducted a Morris water maze test, which can assess hippocampal-dependent spatial long-term memory ability [21]. The scopolamine-treated group showed unchanged escape latency over 4 days. From the second day, administration of 50 and 100 mg/kg of FCL led to significant reductions in the escape latency compared with the scopolamine-treated group (Fig. 3b, *P* < 0.01). Groups treated with 200 mg/kg FCL and donepezil significantly reduced escape latency from the first day (*P* < 0.01). Analysis of the escape latency revealed a significant difference between groups [F(3168) = 28.5, *P* < 0.001], training days [F(5168) = 133.1, *P* < 0.001], and the effects of interaction [F(15168) = 34.28, *P* < 0.001].

On the day following the last training trial sessions (probe trial test), swimming times within the target quadrant in the scopolamine-treated groups were significantly lower than those in the vehicle-treated control group (Fig. 3c, *P* < 0.01). In addition, the reduced swimming time in the target quadrant was reversed by administration of FCL (100 and 200 mg/kg) and donepezil (Fig. 3c, *P* < 0.05). Thus, there were significant group difference in the swimming time [F(5,41) = 6.981, *P* < 0.001]. The results of the behavioral tests suggest that FCL pretreatment improved the long-term memory in the scopolamine-induced amnesia mouse model.

### Inhibitory effect of FCL on AChE activity in vitro

AChE is known to hydrolyze and inactivate ACh, a major excitatory neurotransmitter. Increased AChE activity leads to a decreased level of ACh and thus neurological diseases associated with cholinergic deficits as observed in AD patients [7]. Previous studies have reported that scopolamine increases AChE activity in both the hippocampus and cortex [35]. Accordingly, AChE inhibitors maintain normal ACh levels, resulting in formation of long-term memory and retention of existing memories [36, 37].

**Fig. 4:**
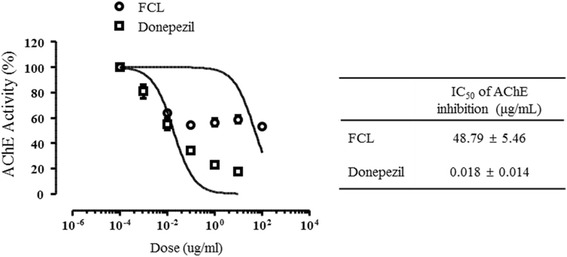
Inhibitory effects of FCL or donepezil on AChE activity in vitro AChE activities were measured using acetylcholine iodide as a synthetic substrate in a colorimetric assay. Each AChE activity was observed in three times. AChE inhibition is expressed as mean ± SEM.

Thus, we investigated whether the memory enhancing effects of FCL as shown by the mice behavioral tests were caused by inhibition of AChE activity. Herein, the in vitro AChE activity was inhibited by FCL in a concentration-dependent manner with an IC_50_ value of 48.79 ± 5.46 μg/ml. The IC_50_ value of donepezil was 0.018 ± 0.014 μg/ml (Fig. 4). These results indicate that the anti-amnestic effect of FCT is mediated through the suppression of AChE in the brain.

### Effect of FCL on scopolamine-attenuated pCREB and BDNF expression in the hippocampus

The effects of FCL on the expression of pCREB and BDNF, which are critical molecules in memory formation, were investigated by immunohistochemical and Western blot analysis using mouse brain tissues. Previous studies have confirmed that pCREB and BDNF are key molecules involved in memory formation, and that activation of CREB transcriptional activity regulates BDNF expression to induce cognitive function [38, 39]. These findings indicated that the CREB signaling pathway is involved in memory enhancement, and that down-regulation of CREB activation leads to deficits in long-term memory. It was reported that mitogen-activated protein kinase (MEK) can phosphorylate extracellular signal-regulated kinase (ERK), and this MEK/ERK pathway is mainly involved in the memory enhancing effects of BDNF. [40].

**Fig. 5:**
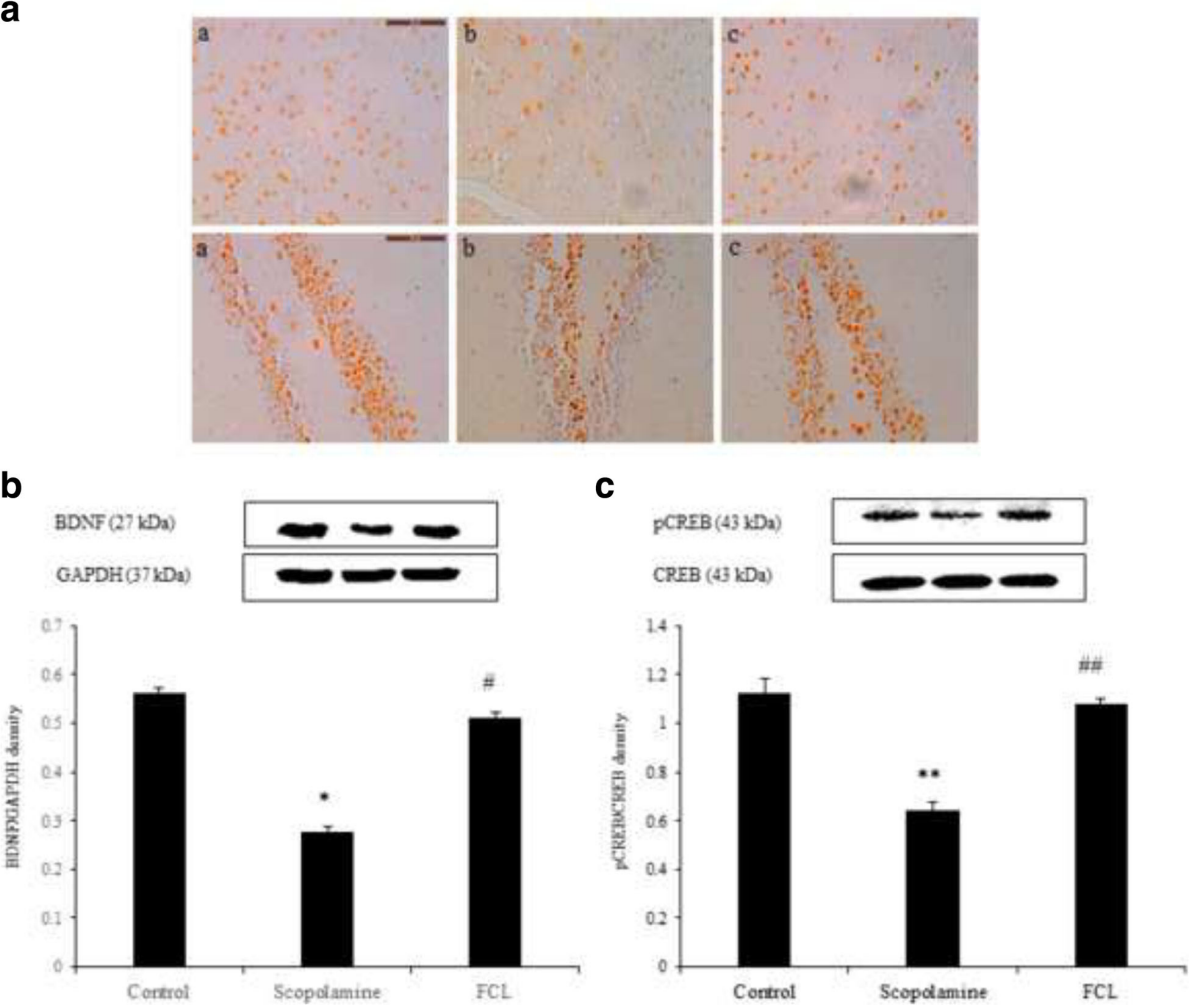
Effects of FCL on pCREB and BDNF in cortex and hippocampus of scopolamine-induced memory deficit. Photomicrographs (**a**) showing the pCREB immunoreactivity in sections of cortex and the dentate gyrus region of hippocampus. Original magnification was 40× **(a-c**). Scale bar = 50 μm. The expression levels were measured by Western blot analysis (**b, c**). The relative expression levels of BDNF (27 kDa) and pCREB (43 kDa) were determined by densitometry and normalized by internal controls, GAPDH (37 kDa) and CREB (43 kDa). Data represent means ± SEM (*n* = 3). * *P* < 0.05, ** *P* < 0.01 as compared with the control group, # *P* < 0.05, ## *P* < 0.01 as compared with the scopolamine group

Scopolamine reduced CREB activation in the cortex and hippocampus. However, FCL administration (200 mg/kg) increased the number of pCREB positive cells in the hippocampal dentate gyrus regions (Fig. 5a). Moreover, the results from Western blot analysis revealed that the hippocampal pCREB and BDNF expressions in mice pretreated with FCL (200 mg/kg) were significantly higher than those in the scopolamine-treated control mice (Fig. 5b, c, *P* < 0.05). These results demonstrate that FCL exerts a memory enhancing effect through the regulation of CREB and BDNF expression. Although FCL reversed the decrease of pCREB and BDNF expression in the hippocampus, the effect of FCL on the molecules involved in the pCREB and BDNF upstream/downstream signaling pathway remains unclear. Thus, further studies are needed to clarify the role of FCL in the CREB signaling pathway.

### Quantification of curcuminoids in FCL by using LC-MS/MS

The two major types of compounds in *C. longa* are curcuminoids and sesquiterpenoids [41–43]. Curcuminoids, mainly curcumin, DMC, and BDMC are yellowish pigments that exhibit diverse biological activities [44], and may be effective for the prevention and treatment of AD [45]. Sesquiterpenoids including ar-tumerone, α-turmerone, β-turmerone and curlone are components of the essential oil of *C. longa*, and have hypoglycemic [41], mosquitocidal [46] and anti-inflammatory activities [47]. In spite of their various health benefits, these active compounds have low bioavailability. Several studies have suggested that vanillin, vanillic acid and ferulic acid are the degradation products of curcumin by exposure to heat or a neutral-alkaline pH environment [48, 49]. Curcuminoids were successfully produced via caffeic acid using on artificial pathway in *Escherichia coli* [50].

Despite its poor bioavailability, curcumin is considered as the most potent and active compound in *C. longa*, and is used as a marker for quality control of functional foods containing *C. longa*. Thus, curcumin, DMC, and BDMC were quantified in FCL by LC-MS/MS in the positive SIM mode. The mixed solution of standards showed major peaks at m/z of 369.2, 339.0, and 309.5, which were assigned to the [M + H]^+^ ions of curcumin, DMC, and BDMC, respectively. Quantitative analysis was performed in the SIM mode. As shown in Table 1, the amounts of curcumin, DMC, and BDMC in freeze-dried powder of FCL were 10.37, 1.68, and 2.33 μg/mg, respectively. The total amount of curcuminoids in FCL was 1.44% (14.38 μg/mg), being lower than the known amount in *C. longa* (2–5%). Nevertheless, FCL may have several advantages such as an increased solubility and stability of curcumin in lactic acid produced by fermentation, as previously reported [51], and it can also be used as a probiotic material. Further research is needed to clearly understand the molecular mechanisms underlying the protective action of the components present in FCL.

**Table 1:** Curcuminoids contents in FCL

Compound	Content (μg/mg)
Curcumin	10.37 ± 0.57
Demothoxycurcumin	1.68 ± 0.08
Bisdemethoxycurcumin	2.23 ± 0.22

Data presented as mean ± SD (*n* = 3)

## Conclusions

In this study, the effectiveness of FCL against memory dysfunction was investigated using oxidative stress-induced cell death in C6 glioma cells, proinflammatory-activated BV2 microglial cells, and the scopolamine-induced amnesia model in mice. Our results demonstrate for the first time that FCL inhibited the cell damage induced by t-BHP and H_2_O_2_ in C6 cells, as well as the production of pro-inflammatory mediators including NO, TNF-α, PGE2, iNOS, and COX-2 in LPS-stimulated BV2 cells. Moreover, FCL improved the learning behavior of mice subjected to scopolamine-induced memory impairment in the step-through passive avoidance test and the water maze test. The memory improving effect of FCL was found to be closely related to the in vitro AChE inhibition, CREB activation, and BDNF expression in the hippocampus. These findings show that the enhancing effects of FCL manufactured through a fermentation process using *L. plantarum* sp. were the result of the inhibition of AChE activity, as well as promotion of the CREB activation and BDNF expression. Moreover, the results of this study provide pharmacological evidence that FCL could be used to alleviate memory impairment.

## Abbreviations

ACh: Acetylcholine
AChE: Acetylcholinesterase
AD: Alzheimer’s disease
BDMC: Bisdemethoxycurcumin
BDNF: Brain-derived neurotropic factor
CNS: Central nervous system
COX-2: Cyclooxygenase-2
CREB: cAMP response element-binding protein
DMC: Demethoxycurcumin
FCL: Fermented *Curcuma. longa*
iNOS: Inducible NO synthase
LC-MS/MS: Liquid chromatography-tandem mass spectrometry
LPS: Lipopolysaccharide
MTT: 3-(4,5-dimethylthiazol-2-yl)-2,5-diphenyl tetrazolium bromide
NO: Nitric oxide
p-CREB: Phosphorylated cAMP response element-binding protein
PGE_2_: Prostaglandin E_2_
t-BHP: Tert-butyl hydroperoxide
TNF-α: Tumor necrosis factor α
